# Preferences for Artificial Intelligence Clinicians Before and During the COVID-19 Pandemic: Discrete Choice Experiment and Propensity Score Matching Study

**DOI:** 10.2196/26997

**Published:** 2021-03-02

**Authors:** Taoran Liu, Winghei Tsang, Yifei Xie, Kang Tian, Fengqiu Huang, Yanhui Chen, Oiying Lau, Guanrui Feng, Jianhao Du, Bojia Chu, Tingyu Shi, Junjie Zhao, Yiming Cai, Xueyan Hu, Babatunde Akinwunmi, Jian Huang, Casper J P Zhang, Wai-Kit Ming

**Affiliations:** 1 Department of Public Health and Preventive Medicine School of Medicine Jinan University Guangzhou China; 2 International School Jinan University Guangzhou China; 3 Faculty of Social Sciences University of Southampton Southampton United Kingdom; 4 Department of Applied Mathmatics The Hong Kong Polytechnic University Hong Kong Hong Kong; 5 College of Computer Science and Technology Henan Polytechnic University Henan China; 6 School of Applied Mathematics Beijing Normal University (Zhuhai) Zhuhai China; 7 Department of Obstetrics and Gynecology Brigham and Women’s Hospital Boston, MA United States; 8 Center for Genomic Medicine, Massachusetts General Hospital Harvard Medical School Harvard University Boston, MA United States; 9 Department of Epidemiology and Biostatistics School of Public Health Imperial College London London United Kingdom; 10 School of Public Health The University of Hong Kong Hong Kong Hong Kong

**Keywords:** propensity score matching, discrete latent traits, patients’ preferences, artificial intelligence, COVID-19, preference, discrete choice, choice, traditional medicine, public health, resource, patient, diagnosis, accuracy

## Abstract

**Background:**

Artificial intelligence (AI) methods can potentially be used to relieve the pressure that the COVID-19 pandemic has exerted on public health. In cases of medical resource shortages caused by the pandemic, changes in people’s preferences for AI clinicians and traditional clinicians are worth exploring.

**Objective:**

We aimed to quantify and compare people’s preferences for AI clinicians and traditional clinicians before and during the COVID-19 pandemic, and to assess whether people’s preferences were affected by the pressure of pandemic.

**Methods:**

We used the propensity score matching method to match two different groups of respondents with similar demographic characteristics. Respondents were recruited in 2017 and 2020. A total of 2048 respondents (2017: n=1520; 2020: n=528) completed the questionnaire and were included in the analysis. Multinomial logit models and latent class models were used to assess people’s preferences for different diagnosis methods.

**Results:**

In total, 84.7% (1115/1317) of respondents in the 2017 group and 91.3% (482/528) of respondents in the 2020 group were confident that AI diagnosis methods would outperform human clinician diagnosis methods in the future. Both groups of matched respondents believed that the most important attribute of diagnosis was accuracy, and they preferred to receive combined diagnoses from both AI and human clinicians (2017: odds ratio [OR] 1.645, 95% CI 1.535-1.763; *P*<.001; 2020: OR 1.513, 95% CI 1.413-1.621; *P*<.001; reference: clinician diagnoses). The latent class model identified three classes with different attribute priorities. In class 1, preferences for combined diagnoses and accuracy remained constant in 2017 and 2020, and high accuracy (eg, 100% accuracy in 2017: OR 1.357, 95% CI 1.164-1.581) was preferred. In class 2, the matched data from 2017 were similar to those from 2020; combined diagnoses from both AI and human clinicians (2017: OR 1.204, 95% CI 1.039-1.394; *P*=.011; 2020: OR 2.009, 95% CI 1.826-2.211; *P*<.001; reference: clinician diagnoses) and an outpatient waiting time of 20 minutes (2017: OR 1.349, 95% CI 1.065-1.708; *P*<.001; 2020: OR 1.488, 95% CI 1.287-1.721; *P*<.001; reference: 0 minutes) were consistently preferred. In class 3, the respondents in the 2017 and 2020 groups preferred different diagnosis methods; respondents in the 2017 group preferred clinician diagnoses, whereas respondents in the 2020 group preferred AI diagnoses. In the latent class, which was stratified according to sex, all male and female respondents in the 2017 and 2020 groups believed that accuracy was the most important attribute of diagnosis.

**Conclusions:**

Individuals’ preferences for receiving clinical diagnoses from AI and human clinicians were generally unaffected by the pandemic. Respondents believed that accuracy and expense were the most important attributes of diagnosis. These findings can be used to guide policies that are relevant to the development of AI-based health care.

## Introduction

Artificial intelligence (AI) technology, which is also called machine intelligence technology, has been used in various fields, such as automation, language, image understanding and analysis, and genetic algorithm research. AI technology can perform better than a human when it comes to performing particular tasks, and such technology has the potential to replace several traditional human occupations. This is the result of continuous advances in medicine, neuroscience, robotics, and statistics. In the medical and health care field [1], AI technology has many widespread applications, and the use of such technology has resulted in a wide range of opportunities for the future. For instance, machine learning technology has been used to analyze medical big data and electronic health records, conduct computer vision research, facilitate natural language processing, and develop intelligent robots [2]. In addition, AI technology has helped address the masses’ demands for increasing the number of clinician services [3].

As of November 13, 2020, the novel COVID-19 disease has spread in over 217 countries [4] and territories across the world. The disease has also resulted in tremendous threats and challenges to public health security systems worldwide. The COVID-19 outbreak has pushed the medical systems and resources of numerous countries to the brink of collapse. Diagnostic AI technology, which includes diagnostic machine learning technology, has started to play a role in relieving the burden that the pandemic has placed on the public health system and easing the shortage of medical resources. At the start of the COVID-19 outbreak, the medical AI team of the Alibaba Academy for Discovery, Adventure, Momentum, and Outlook rapidly developed a set of AI diagnostic technologies that could interpret the computed tomography images of patients with suspected COVID-19 (ie, new COVID-19 cases) within 20 seconds, with an accuracy of 96% [5]. In the fight against the epidemic [6], digital technologies such as cloud computing, artificial intelligence, and blockchain technologies have played a vital role.

The combination of AI technology and human clinician–operated convolutional neural networks [7] has greatly improved the efficiency and accuracy of diagnosis methods and substantially reduced diagnosis times and outpatient queuing times. In 2014, app developers from around the world made a total of US $663.8 million by selling AI health care apps, and their revenue is expected to reach US $666.2 million in 2021 [8]. However, there are various uncertainties with regard to preferences for different diagnostic methods among patients (ie, men and women) from high-income areas and low-income areas in China. Furthermore, there have been no studies that assess patients’ preferences for AI clinicians and human clinicians before and during the COVID-19 pandemic period, and analyze the aspects of patients’ decision-making behaviors during different periods of time.

This study aimed to compare people’s preferences for AI diagnoses and traditional diagnoses (ie, human clinicians’ diagnoses) before and during the COVID-19 pandemic. We assessed two groups of respondents with similar demographic characteristics. We recruited one group in 2017 and the other group in 2020 to learn whether people’s preferences for AI and traditional human clinicians were affected by the pressure of the COVID-19 pandemic. We performed propensity score matching (PSM) to match the two groups. We also conducted a discrete choice experiment (DCE) to quantify and measure peoples’ preferences for different diagnosis methods and identify factors that disrupted and impacted peoples’ decision-making behaviors.

## Methods

### Overview

We designed a web-based questionnaire to collect participants’ demographic information and investigate patients’ preferences for different diagnosis strategies (Multimedia Appendix 1). In brief, the questionnaire included 7 similar hypothetical scenarios. Respondents were asked to choose a preferred diagnosis strategy for each scenario.

We used the PSM method to match two different groups of respondents (ie, the 2017 group and the 2020 group) with similar demographic characteristics. In addition, we used multinomial logit (MNL) models [9,10] and latent class models (LCMs) [11] to evaluate and investigate respondents’ preferences for different diagnosis strategies. We also compared the preferences of the matched respondents from the 2017 group to those of the 2020 group to identify heterogeneity or homogeneity in preferences for diagnosis attributes.

### Selection of Attributes and Levels

Individuals could choose different levels of health care services for each diagnosis attribute. Patients from the outpatient queues of The First Affiliated Hospital of Jinan University (Guangzhou Overseas Chinese Hospital) and The First Affiliated Hospital of Sun Yat-sen University were randomly selected for this study. Each patient was prompted to hypothesize which diagnosis methods or attributes had a large impact on their decision (ie, the methods/attributes that were of prominent importance to each participant).

After assessing patients’ hypotheses and related literature [12-14], we included the following six diagnosis attributes and their respective levels in our questionnaire experiment: (1) diagnostic method (levels: clinician diagnosis, AI and clinician diagnosis, and AI diagnosis); (2) outpatient waiting time before the start of the diagnosis process (levels: 0, 20, 40, 60, 80, and 100 minutes); (3) diagnosis time (levels: 0, 15, and 30 minutes); (4) accuracy (ie, the rate of correct diagnosis; levels: 60%, 70%, 80%, 90%, and 100%); (5) follow-up after diagnosis (ie, whether a doctor can conduct follow-ups at any time; levels: yes or no); and (6) diagnostic expenses (levels: ¥0, ¥50, ¥100, ¥150, ¥200, and ¥250; a currency exchange rate of ¥1=US $0.16 is applicable). Attributes and their respective levels are presented in Textbox 1.

**Diagnosis attributes and their respective levels in this discrete choice experiment.**

**Diagnostic method**

- Description: the diagnosis method that patients prefer
- Levels: clinician diagnosis, artificial intelligence and clinician diagnosis, and artificial intelligence diagnosis

**Outpatient waiting time**

- Description: the amount of time that patients wait in a queue before the diagnosis process
- Levels: 0 minutes, 20 minutes, 40 minutes, 60 minutes, 80 minutes, and 100 minutes

**Diagnosis time**

- Description: the amount of time before a patient obtains a diagnosis
- Levels: 0 minutes, 15 minutes, and 30 minutes

**Diagnostic accuracy**

- Description: the rate of correct diagnosis
- Levels: 60%, 70%, 80%, 90%, and 100%

**Follow-up after diagnosis**

- Description: case tracking and follow-ups after diagnosis
- Levels: Yes and no

**Diagnostic expenses**

- Description: the cost of diagnosis
- Levels: ¥0, ¥50, ¥100, ¥150, ¥200, and ¥250 (a currency exchange rate of ¥1=US $0.16 is applicable)

### DCE Instrument Design and Questionnaire

With regard to the design our DCE instrument, we used the fractional factorial design method [15,16] to identify the optimal number of treatment scenarios. This process was conducted with Lighthouse Studio version 9.8.1 (Sawtooth Software). In practice, it is not always feasible for respondents to choose among all of the possible combinations of attributes and levels (ie, full factorial design). The full factorial design of the DCE instrument had 3240 different combinations (ie, 3 × 6 × 3 × 5 × 2 × 6 = 3264), which is an unreasonable number of options to present to respondents. Thus, the fractional factorial method was essential in designing the DCE instrument. This method is based on the following two principles [15-17]: (1) orthogonality, which, in terms of the DCE, means that each attribute level should have little to no correlation with other attribute levels; and (2) balance, which means that each attribute should appear an equal number of times. After considering these principles, we provided 6 random questions and 1 fixed question to each respondent in the DCE.

The DCE questionnaire contained 2 parts. The first part required the respondents to fill in their demographic information, such as age (ie, 18-20, 21-25, 26-30, 31-35, 36-40, 41-45, 46-50, 51-55, 56-60, 61-65, 66-70, 71-75, 76-80, and 81-85 years), sex (ie, male or female), and educational level (ie, primary school student, primary school graduate, middle school student, middle school graduate, high school student, high school graduate, undergraduate, bachelor’s degree, graduate student, master’s degree, postgraduate student, and doctorate degree). The second part required the respondents to consider seven different scenarios. For each scenario, respondents were to imagine that they were in an outpatient queue waiting for a diagnosis. They were then asked to choose a preferred diagnosis strategy. At the end of the questionnaire, respondents were required to estimate the number of years (ie, 5 years, 10 years, 15 years, 20 years, 30 years, 40 years, or never) it would take for AI clinicians to surpass human clinicians. The scenarios and the options for the different types of clinicians are presented in Multimedia Appendix 2.

### Data Collection

In October 2017 and August 2020, we sent our website link to people of different age groups by using various social media platforms, such as WeChat (Tencent Inc) and QQ (Tencent Inc). People could use the link to access the DCE questionnaire, which was the same for each participant. To increase the response rate, we provided incentives (ie, a lottery for a Fitbit watch and cash prizes) for completing the questionnaire.

At the beginning of the questionnaire, we provided a brief background on the applications of AI in medicine. This included information on the potential advantages and disadvantages of AI clinicians and traditional clinicians, and the purpose of our DCE. The questionnaire only took 5-10 minutes to complete. Respondents had to click the “Agree to take the survey” button to start filling out the questionnaire. Once respondents clicked the “Agree to take the survey” button, they were notified that they willingly chose to participate in this study. Respondents were also notified that their privacy was protected by the law.

### PSM

PSM is a regression method for identifying treatment group and control group patients with similar basic characteristics. This method is prevalently used in the study of impact factors and causal effects, such as those in medical treatments, policy decisions, or case studies. PSM involves the following five steps [18]: (1) estimating propensity scores; (2) choosing a matching algorithm; (3) checking for overlap/common support; (4) estimating the quality and effects of the matching results; and (5) conducting a sensitivity analysis. The mathematical theory for PSM is primarily based on the Roy-Robin model [19-21]. Our objective was to perform a PSM analysis in which participants who were recruited in 2017 were treated as the treatment group, and participants who were recruited in 2020 were treated as the control group. Participants’ PSM data are provided in Multimedia Appendix 3 [18]. We matched the respondents in each group according to their demographic characteristics, such as age, sex, and educational level. All demographic information was coded as dummy variables; for instance, male respondents were coded as “1,” and female respondents were coded as “0.”

### Matching Algorithm

Although there are various matching algorithms [18], we used the nearest neighbor [22] algorithm because it was appropriate for identifying individuals in one group that best matched the individuals in another group. Another merit of the nearest neighbor algorithm is that it can differentiate between individuals in the control group and individuals in the treatment group, which guarantees that all treated individuals are successfully matched. Therefore, the nearest neighbor algorithm provides the most information on treatment groups and control groups. Additionally, we conducted a 1:1 matching analysis, which effectively reduces confounding bias [23] and improves research efficiency and credibility.

### Statistical Analysis

#### MNL Model

There are various analysis models that can be used to conduct DCE-related statistical analyses, such as random effects binary probit and logit models, MNL models, and mixed logit models [16,24]. The theoretical model for a DCE is based on the random utility model (Multimedia Appendix 4) [16]. We assumed that respondents’ choices would maximize the utility of each question in the DCE questionnaire. The overall utility of decision makers is based on fixed utility and random utility, which are unobservable. We assessed respondents’ preferences by analyzing their comments. This allowed us to identify random utilities that could not be identified by analyzing a question.

We used the MNL model to analyze people’s preferences for different attribute levels. Our independent variable only accounted for attributes that were related to health care plans; it did not account for any information that was related to participants. The MNL model was used to analyze respondents’ health care plans, which were chosen based on the relative importance of the plans’ attributes and the “none” option. The coded value of each participants’ chosen health care plan was calculated based on participants’ coded responses to questions about queuing times, diagnosis times, and diagnostic costs. We used a maximum likelihood approach to analyze MNL model data.

The results from the MNL model were determined by the options for health care plans, as the data for this attribute were grouped before analysis. In the MNL model, “effect” is synonymous with “utility.” Therefore, positive MNL model coefficients indicated that individuals preferred one level of service over other levels for the same attribute. The MNL model in this study was based on a similar logistic regression model. The MNL model–based observations correlated with those in blocks that corresponded with the same individual. Instead of having 1 level line per individual like in the classical logit model, the MNL model had 1 level line per attribute level of interest (ie, for each individual). For example, in this study, we analyzed three types of diagnoses (ie, clinician diagnoses, AI and clinician diagnoses, and AI diagnoses), and each type had its own characteristics. However, an individual could only choose 1 of the 3 types of diagnoses. As per the characteristics of the MNL model, all three options were presented to each respondent, and all respondents could choose their preferred option. We reported the odds ratios (ORs) of respondents’ preferences for different attribute levels.

#### LCM

We used an LCM [11] to create different classes for individuals with similar preferences. The purpose of the LCM was to identify correlations among explicit variables, create the fewest number of classes, and achieve local independence. An LCM initially assumes that the null model is the hypothesized model and that local independence exists among explicit variables. Afterward, the LCM increases the number of latent categories in the null model and uses a maximum likelihood approach to create various models, which are based on parameters’ limitations. The LCM then tests the hypothesized model and observed data, compares the hypothesized model to the other models, and identifies the most appropriate model. Although there are different types of model information evaluation criteria, Akaike information criteria [25] and Bayesian information criteria [26] are the most prevalently used criteria for selecting LCMs. After the model was created, observed data were classified into the appropriate latent classes.

#### Willingness to Pay

Willingness to pay (WTP) is an efficient metric for measuring how much an individual is willing to sacrifice (ie, economic sacrifices) to choose one diagnosis attribute level over another (ie, the reference attribute level). We analyzed participants’ WTP to identify homogeneity and heterogeneity in participants’ preferences.

### Software

Propensity score matching was conducted with Stata 16 (StataCorp LLC), and the MNL model and LCMs were created with Lighthouse Studio version 9.8.1 (Sawtooth Software).

## Results

### Data Collection

Of the 1520 individuals who visited our DCE website in 2017, 1317 (86.6%) completed the questionnaire and were included in the analysis. Of these 1317 respondents, 1317 (100%) were aged 18-85 years, 731 (55.5%) were female, and 1115 (84.7%) believed that AI clinicians would surpass or replace human clinicians.

Of the 874 individuals who visited our new DCE website in 2020, 528 (60.4%) completed the questionnaire. Of these 528 participants, 272 (51.5%) were female and 482 (91.3%) were confident that AI diagnoses were better than traditional diagnoses.

### General PSM and MNL Model Results

**Figure 1:**
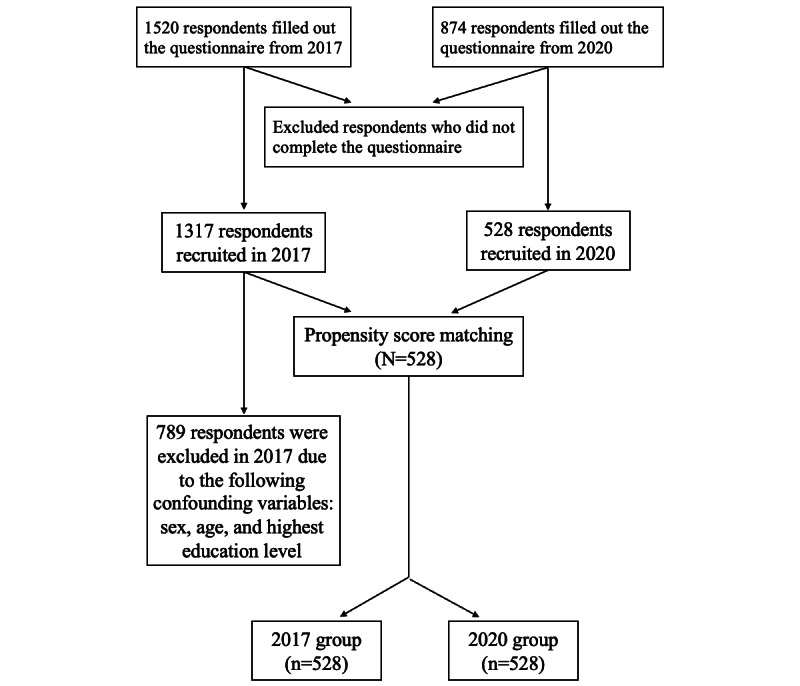
Propensity score matching procedure.

**Figure 2:**
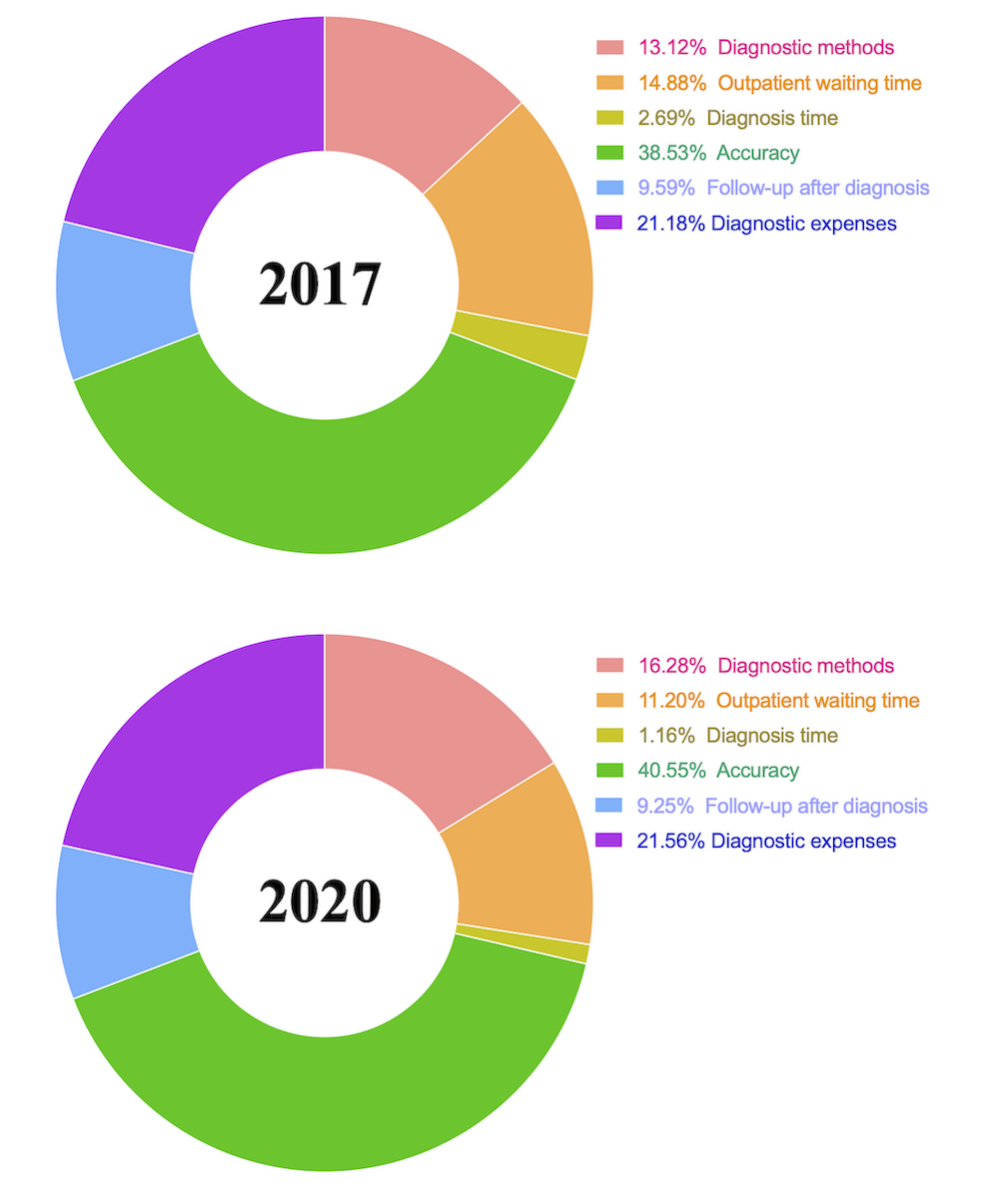
General estimated weighted importance of diagnosis attributes in 2017 and 2020.

Of the 1317 respondents who were recruited in 2017, 528 (40.1%) were matched (ie, via PSM) to the 528 respondents who were recruited in 2020. The PSM procedure is presented in Figure 1, and the demographic characteristics of respondents before and after PSM are presented in Table 1. The general MNL model results for the 2017 and 2020 groups are presented in Table 2, which shows estimated average preference weights (ie, effect weights), *P* values, ORs, and 95% confidence intervals. Generally, individuals in the 2017 and 2020 groups believed that accuracy was the most important diagnosis attribute (Figure 2). The weighted importance value of accuracy was 38.53% in the 2017 group and 40.55% in the 2020 group. Respondents believed that diagnosis time was the least important attribute (weighted importance in 2017: 2.69%; weighted importance in 2020: 1.16%). Additionally, individuals in the 2017 and 2020 groups preferred to receive combined diagnoses from both AI and human clinicians over AI-only diagnoses or human clinician–only diagnoses (2017: OR 1.645, 95% CI 1.535-1.763; 2020: OR 1.513, 95% CI 1.413-1.621; reference: clinician diagnosis; Table 2). In addition, the ORs for the levels of diagnosis accuracy increased as the accuracy increased, which indicated that people will always prefer diagnosis methods with high accuracy. For instance, in the 2017 group, 100% accuracy had an OR of 5.043 (95% CI 4.534-5.609). In the 2020 group, 100% accuracy had an OR of 5.263 (95% CI 4.734, 5.852). The preferences of the matched respondents in the 2017 group were very similar to those of the respondents in the 2020 group.

**Table 1:** Demographic characteristics of nonmatched and propensity score–matched respondents.

Baseline matching characteristics		Nonmatched respondents			Propensity score–matched respondents		
		2017 group (n=1317), n (%)	2020 group (n=528), n (%)	*P* value	2017 group (n=528), n (%)	2020 group (n=528), n (%)	*P* value
**Sex**				<.001			.97
	Male	586 (44.5)	256 (48.48)		250 (47.35)	256 (48.48)	
	Female	731 (55.5)	272 (51.52)		278 (52.65)	272 (51.52)	
**Age (years)**				<.001			.69
	<35	1106 (83.98)	348 (65.91)		379 (71.78)	348 (65.91)	
	≥35	211 (16.02)	180 (34.09)		149 (28.22)	180 (34.09)	
**Highest education level**				<.001			.13
	Primary school graduate to undergraduate	1033 (78.44)	336 (63.64)		385 (72.92)	336 (63.64)	
	Bachelor’s degree to doctorate degree	284 (21.56)	192 (36.36)		143 (27.08)	192 (36.36)	

**Table 2:** General results of the multinomial logit model. Data on propensity score–matched respondents’ preferences for diagnosis attributes in 2017 and 2020 are reported (N=528).

Attributes and levels			2017 group						2020 group				
			Effect coefficient		*P* value		Odds ratio (95% CI)		Effect coefficient		*P* value		Odds ratio (95% CI)
**Diagnosis method**													
	Clinician	−0.15		<.001		Reference		−0.05		.12		Reference	
	Artificial intelligence and clinician	0.35		<.001		1.64 (1.535-1.763)		0.36		<.001		1.51 (1.413-1.621)	
	Artificial intelligence	−0.20		<.001		0.95 (0.885-1.016)		−0.31		<.001		0.78 (0.725-0.833)	
**Outpatient waiting time (minutes)**													
	0	0.31		<.001		Reference		0.15		.01		Reference	
	20	0.12		.03		0.82 (0.741-0.914)		0.26		<.001		1.12 (1.013-1.245)	
	40	−0.03		.57		0.71 (0.639-0.789)		−0.02		.72		0.85 (0.762-0.942)	
	60	−0.08		.12		0.67 (0.606-0.748)		−0.20		<.001		0.71 (0.640-0.788)	
	80	−0.31		<.001		0.54 (0.482-0.595)		−0.20		<.001		0.71 (0.640-0.789)	
**Diagnosis time (minutes)**													
	0	0.05		.19		Reference		−0.02		.57		Reference	
	15	−0.07		.06		0.89 (0.834-0.957)		−0.01		.83		1.01 (0.946-1.084)	
	30	0.02		.53		0.98 (0.912-1.046)		0.03		.43		1.05 (0.980-1.122)	
**Diagnosis accuracy (% accuracy)**													
	60	−0.83		<.001		Reference		−0.83		<.001		Reference	
	70	−0.35		<.001		1.62 (1.458-1.802)		−0.41		<.001		1.52 (1.365-1.684)	
	80	0.07		.16		2.47 (2.235-2.737)		−0.02		.72		2.25 (2.033-2.487)	
	90	0.32		<.001		3.18 (2.867-3.526)		0.43		<.001		3.51 (3.169-3.891)	
	100	0.79		<.001		5.04 (4.534-5.609)		0.83		<.001		5.26 (4.734-5.852)	
**Follow-up after diagnosis**													
	Yes	0.20		<.001		Reference		0.19		<.001		Reference	
	No	−0.20		<.001		0.67 (0.620-0.698)		−0.19		<.001		0.69 (0.656-0.715)	
**Diagnosis expenses (¥^a^)**													
	0	0.42		<.001		Reference		0.36		<.001		Reference	
	50	0.28		<.001		0.87 (0.769-0.976)		0.23		<.001		0.88 (0.782-0.989)	
	100	−0.01		.82		0.65 (0.576-0.730)		0.18		<.001		0.83 (0.738-0.935)	
	150	0.03		.66		0.67 (0.599-0.760)		−0.06		.30		0.65 (0.580-0.736)	
	200	−0.24		<.001		0.52 (0.459-0.585)		−0.19		<.001		0.58 (0.510-0.648)	
	250	−0.47		<.001		0.41 (0.363-0.465)		−0.52		<.001		0.41 (0.366-0.468)	

^a^A currency exchange rate of ¥1=US $0.16 is applicable.

### Overall WTP

In 2017, respondents were willing to pay ¥13.99 to receive combined diagnoses from AI and human clinicians. Additionally, people were not willing to pay for longer outpatient waiting times, but they were willing to pay for higher diagnosis accuracy (ie, ¥1.60 per 1% increase in accuracy). In 2020, respondents were willing to pay ¥0.79 to receive combined diagnoses from AI and human clinicians instead of clinician-only diagnoses. Compared to respondents’ WTP for certain diagnosis methods in 2017, respondents’ WTP in 2020 was lower. Furthermore, similar to the 2017 group, respondents in the 2020 group were also not willing to pay for longer outpatient waiting times. However, they were willing to pay for higher diagnosis accuracy.

### LCM Results

After comparing the Akaike information criteria, Bayesian information criteria, and Akaike/Bayesian information criteria of the various potential classes, we chose three classes that were the most appropriate for the matched respondents in the 2017 and 2020 groups. The proportions of matched respondents from the 2017 group in each of the three classes were 43.2% (class 1: 228/528), 42.2% (class 2: 223/528) and 14.6% (class 3: 77/528). The proportions of matched respondents from the 2020 group in each of the three classes were 44.8% (class 1: 237/528), 48.2% (class 2: 254/528) and 7% (class 3: 37/528).

**Figure 3:**
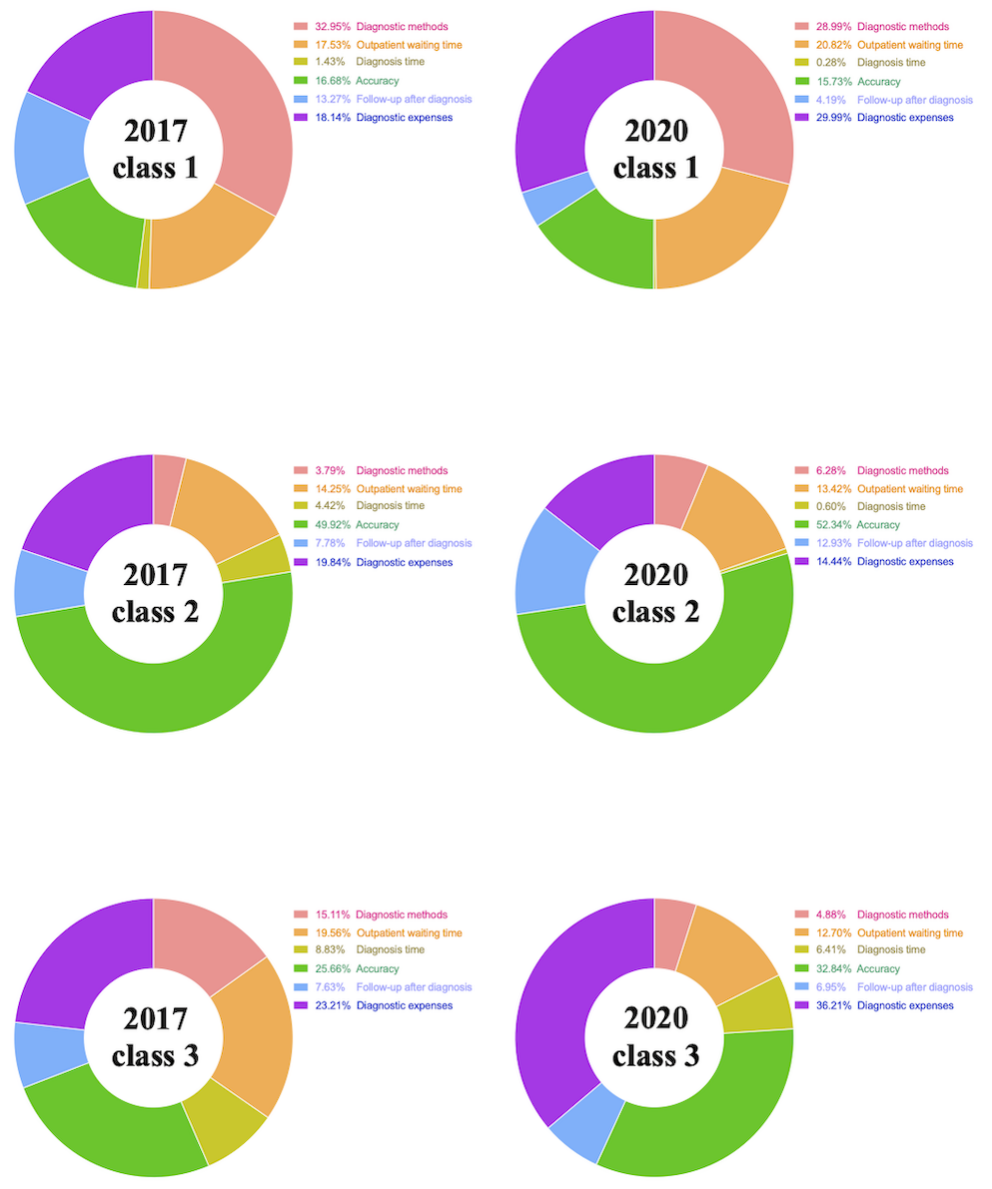
Weighted importance of diagnosis attributes in 2017 and 2020, as determined by the latent class model.

With regard to class 1 (n=228), Figure 3 shows that matched respondents in the 2017 group believed that diagnosis method was the most important attribute (weighted importance: 32.95%), followed by diagnosis expenses (weighted importance: 18.14%). In class 2, matched respondents from the 2017 group believed that diagnosis accuracy (weighted importance: 49.92%) and diagnosis expenses (weighted importance: 19.84%) were the most important attributes. In class 3, matched respondents from the 2017 group believed that diagnosis accuracy (weighted importance: 25.66%) and diagnosis expenses (weighted importance: 23.21%) were the most important attributes. In class 1, the respondents from the 2020 group believed that diagnosis expenses (weighted importance: 29.99%) and diagnosis method (weighted importance: 28.99%) were the most important attributes. In class 2, the respondents from the 2020 group believed that diagnosis accuracy (weighted importance: 52.34%) was the most important attribute, followed by diagnosis expenses (weighted importance: 14.44%). In class 3, the respondents from the 2020 group believed that diagnosis expense (weighted importance: 36.21%) was the most important attribute, followed by diagnosis accuracy (weighted importance: 32.84%). It was obvious that the three factors that respondents believed were the most important were diagnosis accuracy, diagnosis expenses, and diagnosis methods. In some classes, respondents believed that diagnosis method was the most important attribute. However, respondents typically believed that diagnosis accuracy was the most important attribute and diagnosis expense was the second most important attribute.

According to our ORs for classes 1 and 2, the respondents in the 2017 group (Table S1 in Multimedia Appendix 5) preferred the combined diagnosis method (class 1: OR 2.479, 95% CI 0.997-2.743; class 2: OR 1.204, 95% CI 1.039-1.394) over the other two methods. This was not true for respondents in class 3. Respondents in classes 1 and 3 preferred an outpatient waiting time of 0 minutes, and respondents in classes 1 and 2 preferred a diagnosis time of 0 minutes. Respondents across all classes preferred a diagnosis cost of ¥0. Furthermore, respondents in the 2017 group (ie, those in all classes) preferred high diagnosis accuracy (eg, 100% accuracy in class 3: OR 4.899, 95% CI 3.631-6.611). Respondents in all classes believed that follow-ups after diagnosis were important.

**Figure 4:**
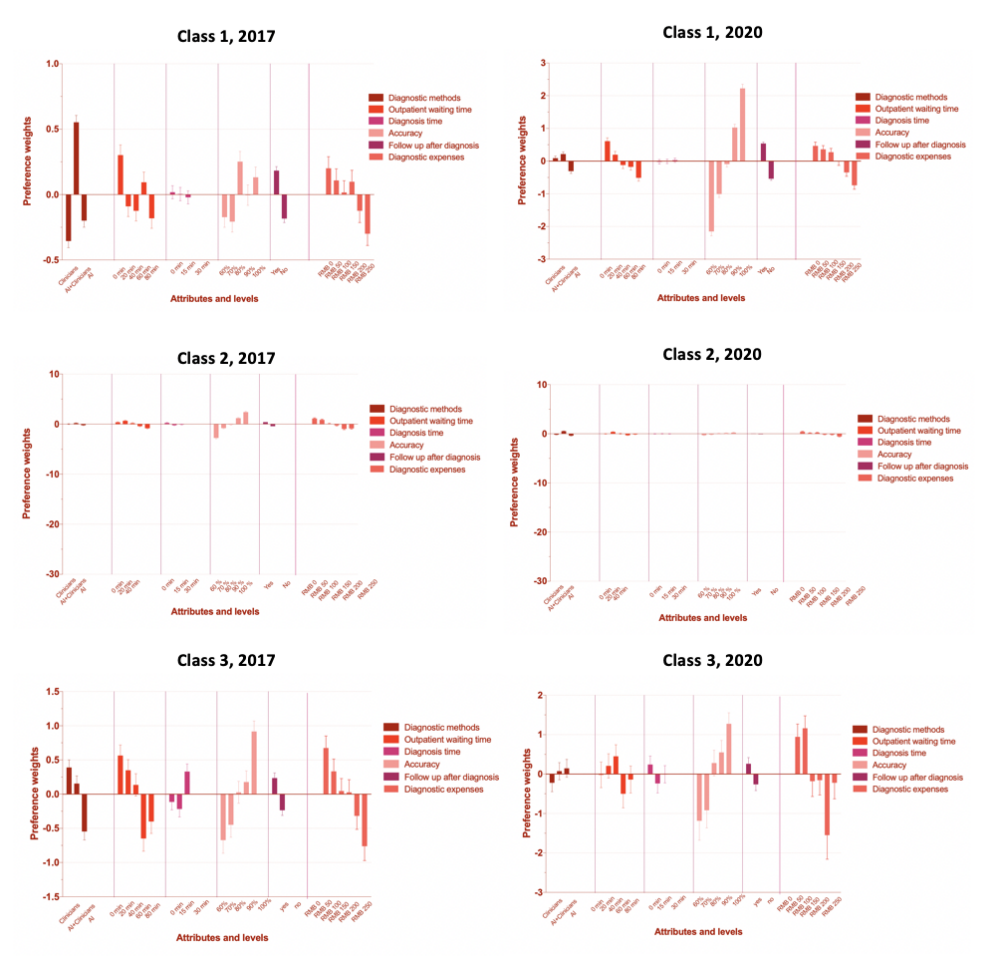
Preference weights stratified by year (ie, 2017 and 2020) and class (ie, classes 1, 2, and 3), as determined by the latent class model.

In classes 1 and 2, the respondents from the 2020 group (Table S2 in Multimedia Appendix 5) preferred the combined diagnosis method (class 1: OR 1.135, 95% CI 0.997-1.293; class 2: OR 2.009, 95% CI 1.826-2.211). This was not true for class 3. Respondents in class 2 preferred an outpatient waiting time of 20 minutes (OR 1.488, 95% CI 1.287-1.721). Additionally, similar to the 2017 group, the respondents in the 2020 group (ie, those in all classes) preferred high accuracy. Follow-ups after diagnosis were important to the respondents in the 2020 group (ie, those in all classes). The strength of respondents’ preferences is visually presented in Figure 4; preference strength was quantified by calculating the preference weight (ie, coefficient) of each attribute’s level.

We found that respondents’ WTP was highly consistent with the corresponding ORs of each attribute. In classes 1 and 2, the respondents from the 2017 group (Table 3) were willing to pay for the combined diagnosis method. This was not true for class 3. Additionally, in class 3, the respondents from the 2017 group were the only respondents who were willing to pay for longer diagnosis times. The respondents from the 2017 group (ie, those in all classes) were willing to pay for higher diagnosis accuracy and follow-ups after diagnosis.

In classes 1 and 2, the respondents from the 2020 group (Table 4) were willing to pay for the combined diagnosis method. This was not true for class 3, in which respondents were willing to pay more for the AI diagnosis method. The respondents from the 2020 group (ie, those in all classes) were willing to pay for shorter outpatient waiting times, higher diagnosis accuracy, and follow-ups after diagnosis.

**Table 3:** Respondents’ WTP^a^ in 2017.^b^

Attribute		Overall WTP (N=528), ¥ (US $)	WTP in class 1 (n=228), ¥ (US $)	WTP in class 2 (n=223), ¥ (US $)	WTP in class 3 (n=77), ¥ (US $)
**Diagnosis method**					
	Artificial intelligence and clinician	−13.99 (−2.24)	−3.03 (−0.48)	−0.22 (−0.04)	0.31 (0.05)
	Artificial intelligence	1.50 (0.24)	−0.52 (−0.08)	0.25 (0.04)	1.22 (0.20)
Outpatient waiting time		8.92 (1.43)	0.62 (0.10)	0.96 (0.15)	0.53 (0.09)
Diagnosis time		−0.57 (−0.09)	0.07 (0.01)	0.07 (0.01)	−0.44 (−0.07)
Diagnosis accuracy		−1.14 (−0.18)	−0.44 (−0.07)	−2.85 (−0.46)	−1.20 (−0.19)
Follow-up after diagnosis		11.32 (1.81)	1.22 (0.20)	0.95 (0.15)	0.62 (0.10)
Diagnosis expenses		Reference	Reference	Reference	Reference

^a^WTP: willingness to pay.

^b^Negative currency values refer to the amount that respondents were willing to pay for another level.

**Table 4:** Respondents’ WTP^a^ in 2020.^b^

Attribute		Overall WTP (N=528), ¥ (US $)	WTP in class 1 (n=237), ¥ (US $)	WTP in class 2 (n=254), ¥ (US $)	WTP in class 3 (n=37), ¥ (US $)
**Diagnosis method**					
	Artificial intelligence and clinician	−0.79 (−0.13)	−0.17 (−0.03)	−1.33 (−0.21)	−1.31 (−0.21)
	Artificial intelligence	0.48 (0.07)	0.54 (0.09)	0.42 (0.07)	−1.62 (−0.26)
Outpatient waiting time		0.38 (0.06)	0.70 (0.11)	0.19 (0.03)	0.61 (0.10)
Diagnosis time		−0.05 (−0.01)	−0.04 (−0.01)	0.004 (0.001)	0.06 (0.01)
Diagnosis accuracy		−1.60 (−0.26)	−3 (−0.48)	−0.44 (−0.07)	−5.65 (−0.90)
Follow-up after diagnosis		0.73 (0.12)	1.46 (0.23)	0.25 (0.04)	2.31 (0.37)
Diagnosis expenses		Reference	Reference	Reference	Reference

^a^WTP: willingness to pay.

^b^Negative currency values refer to the amount that respondents were willing to pay for another level.

**Figure 5:**
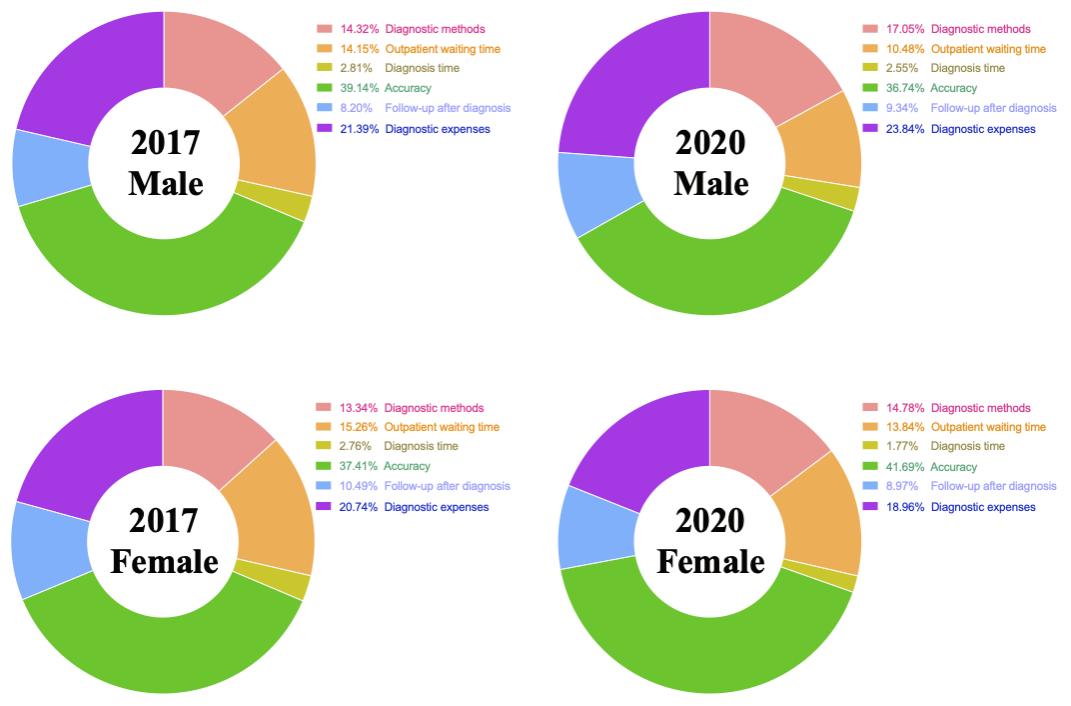
Weighted importance of diagnosis attributes in 2017 and 2020, as determined by the latent class model, which stratified data according to sex (ie, male and female respondents).

According to the LCM, which stratified data according to sex, male respondents in the 2017 group (Figure 5) believed that the most important attribute was diagnosis accuracy (weighted importance: 39.14%), followed by diagnosis expenses (weighted importance: 21.39%). Female respondents in the 2017 group also thought that diagnosis accuracy (weighted importance: 37.41%) and diagnosis expenses (weighted importance: 20.74) were the most important attributes. Male respondents in the 2020 group thought that diagnosis accuracy (weighted importance: 36.74%) was the most important attribute, followed by diagnosis expenses (weighted importance: 23.84%). Additionally, female respondents in the 2020 group believed that diagnosis accuracy (weighted importance: 41.69%) was the most important attribute, followed by diagnosis expenses (18.96%). The LCM for male and female respondents in the 2017 and 2020 groups showed that there was no obvious heterogeneity among these respondents’ preferences.

## Discussion

### Principal Results

In this study, we collected information on people’s preferences for AI-based diagnosis by analyzing two different groups of individuals who were recruited in 2017 and 2020 (ie, before and during the COVID-19 pandemic). We used the PSM method to match two groups of respondents with similar demographic characteristics (ie, age, sex, and educational level). After comparing the demographically similar respondents in the 2017 and 2020 groups, we did not find any substantial differences in respondents’ preferences. Diagnosis accuracy and diagnosis expenses were the most important factors that influenced respondents’ preferences.

The success of a DCE questionnaire always depends on the response rate. In other words, people who actively click the website link and complete the questionnaire are essential for expanding sample sizes and the scope of a study. By using the PSM method, we were able to easily assess whether people’s preferences during normal times changed during unusual times (ie, the COVID-19 pandemic).

In this study, we used two different models—the MNL model and the LCM. Both models have various advantages and drawbacks with regard to quantifying respondents’ preferences. According to the general PSM logit model, respondents in both groups consistently believed that accuracy was the most important diagnosis attribute, regardless of their preferences for diagnosis methods. Moreover, diagnosis expense was an important factor that influenced respondents’ decisions in both 2017 and 2020. Respondents believed that this attribute was the second most important attribute. The limited accessibility and availability of medical resources are big problems in China, especially in several rural areas of China. These problems are the result of insufficient medical insurance distribution [27,28] and the country’s low per capita income.

We found that people’s preferences for different diagnoses were largely similar. This indicates that people’s decisions and their preferences for different diagnoses are not considerably affected by pandemic-related factors. However, according to our LCM, there was slight heterogeneity in the preferences of different groups of respondents (eg, male and female respondents). This heterogeneity was not observed in the logit model. Although the weighted importance of accuracy remained consistent across all classes, it might not be the most important factor that affects people’s decisions. In class 1, the respondents from the 2017 and 2020 groups believed that diagnosis expense was the most important factor that affected their decisions, followed by diagnosis method. Based on the LCM results, male respondents in the 2017 and 2020 groups believed that diagnosis accuracy was the most important attribute to consider when choosing a diagnosis strategy.

With regard to attribute levels, we found that respondents typically preferred to receive a combined diagnosis from both AI and human clinicians over a diagnosis from a single source (ie, AI diagnoses or human clinician diagnoses). This is understandable, since respondents typically believed that diagnosis accuracy could be improved by combining different modes of diagnosis. Additionally, it should be noted that several respondents preferred longer diagnosis and outpatient queuing times. Although no studies have reported that diagnosis time and outpatient time correlate with diagnosis accuracy, it is possible that some patients prefer waiting for a doctor over receiving a quicker diagnosis, as they may believe that waiting results in more accurate diagnoses. The low accessibility and high price of AI services are important issues, especially in rural or low-income areas. Therefore, before pricing an AI technology–based service, it is advisable to survey residents and analyze their disposable income. With regard to residents in rural areas, governments should consider adding AI diagnoses to health insurance plans or related subsidy projects. Another AI diagnosis factor that should be considered is accuracy, since companies should only promote and advertise products/services with a high accuracy. When an AI technology–based service enters the market, relevant users should consider combining AI technology with human wisdom during the early stage of market penetration. Therefore, in the future, AI diagnosis technology developers should focus on improving diagnosis accuracy and reducing the cost of diagnoses to make such technology accessible to a wide range of patients.

### Limitations

Our study has several shortcomings and limitations, especially with regard to our data collection process. It was clear that our small sample size limited the power of our analyses. Additionally, our sample might not be representative of the entire Chinese population. Furthermore, the deployment/distribution of AI technology–based medical services is limited, especially in rural areas [29] and areas that consist of uneducated residents. Thus, there are still many obstacles to overcome before AI technology becomes popular; many developments are still needed to popularize conceptual projects.

### Conclusion

Our study shows that respondents’ preferences for AI clinicians in 2017 did not substantially differ from those in 2020. Therefore, people’s preferences for AI diagnoses and clinical diagnoses were unaffected by the COVID-19 pandemic. However, preferences for high diagnostic accuracy and low diagnosis expenses were evident, regardless of people’s preferences for diagnosis methods, waiting times, and follow-up services.

In summary, affordability and accuracy are the two principal factors that should be considered when promoting AI-based health care. The combination of AI-based and professional health care will be more easily accepted by the general public as AI technology develops.

## Appendix

Multimedia Appendix 1 Survey introduction.

Multimedia Appendix 2 Supplementary questionnaire.

Multimedia Appendix 3 Propensity score matching method.

Multimedia Appendix 4 Random utility model.

Multimedia Appendix 5 Supplementary tables.

## Abbreviations

AI: artificial intelligence
DCE: discrete choice experiment
LCM: latent class model
MNL: multinomial logit
OR: odds ratio
PSM: propensity score matching
WTP: willingness to pay
