# Supplementary material for: Preferences for Artificial Intelligence Clinicians Before and During the COVID-19 Pandemic: Discrete Choice Experiment and Propensity Score Matching Study
Source: J Med Internet Res. 2021 Mar 2;23(3):e26997. doi: 10.2196/26997 (PMC7927951; doi:10.2196/26997)
Supplement: Multimedia Appendix 1 [file jmir_v23i3e26997_app1.docx]

**Appendix Survey (Introduction part)**

Artificial intelligence diagnosis and treatment technology has become mature day by day. Many articles related to artificial intelligence in medicine have been published in some famous journals such as *Nature* and *Science*. In the diagnosis of brain tumor pathology, diagnosis of children's cognitive impairment, and diagnosis of skin cancer, the accuracy of AI has exceeded that of the attending doctor s level. In the judgment of objective data indicators, AI has a strong advantage. Its super learning ability, non-fatigue and the ability to continuous work can improve the quality and efficiency of medical services.

When giving patients two choices of artificial intelligence diagnosis and treatment and traditional doctor's diagnosis and treatment, everyone will comprehensively consider various situations and choose the appropriate way of treatment. For example: the accuracy of artificial intelligence diagnosis? Risk of misdiagnosis? Different people have different choices in different situations when choosing the way of medical treatment.

Through this survey, we hope to understand your priority factors when choosing AI diagnosis or traditional doctor diagnosis. Your answers will help doctors and artificial intelligence medical researchers to develop more humane artificial intelligence diagnosis and treatment, and to promote medical development in a better way.

**Survey availability**

The survey can be found at: https://AI2020.sawtoothsoftware.com/login.html
