# Supplementary material for: Preferences for Artificial Intelligence Clinicians Before and During the COVID-19 Pandemic: Discrete Choice Experiment and Propensity Score Matching Study
Source: J Med Internet Res. 2021 Mar 2;23(3):e26997. doi: 10.2196/26997 (PMC7927951; doi:10.2196/26997)
Supplement: Multimedia Appendix 2 [file jmir_v23i3e26997_app2.docx]

**S1 Questionnaire**

**Question 1**

When you have the same disease and the same physical condition, there are three options to choose Doctor A or Doctor B and neither. Please choose the one you are most satisfied with from the following three options?

|  | **Doctor A** | **Doctor B** | **None** |
| --- | --- | --- | --- |
| **Diagnosis method** | Line up for face-to-face visits in outpatient clinics | AI rapid initial diagnosis + doctor's diagnosis |  |
| **Outpatient waiting time** | 80 min | 40 min |  |
| **Diagnosis time** | 15 min | 0 min |  |
| **Accuracy** | 100 % | 70 % |  |
| **Follow-up after diagnosis** | Yes | No |  |
| **Diagnosis expense** | RMB 200 | RMB 250 |  |
| **Which option would you most prefer?**  **(tick one box)** |  |  |  |

**Question 2**

When you have the same disease and the same physical condition, there are three options to choose Doctor A or Doctor B and neither. Please choose the one you are most satisfied with from the following three options?

|  | **Doctor A** | **Doctor B** | **None** |
| --- | --- | --- | --- |
| **Diagnosis method** | Line up for face-to-face visits in outpatient clinics | AI rapid diagnosis |  |
| **Outpatient waiting time** | 40 min | 0 min |  |
| **Diagnosis time** | 15 min | 30 min |  |
| **Accuracy** | 60 % | 80 % |  |
| **Follow-up after diagnosis** | No | Yes |  |
| **Diagnosis expense** | RMB 50 | RMB 100 |  |
| **Which option would you most prefer?**  **(tick one box)** |  |  |  |

**Question 3**

When you have the same disease and the same physical condition, there are three options to choose Doctor A or Doctor B and neither. Please choose the one you are most satisfied with from the following three options?

|  | **Doctor A** | **Doctor B** | **None** |
| --- | --- | --- | --- |
| **Diagnosis method** | AI rapid initial diagnosis + doctor's diagnosis | AI rapid diagnosis |  |
| **Outpatient waiting time** | 0 min | 80 min |  |
| **Diagnosis time** | 30 min | 0 min |  |
| **Accuracy** | 90 % | 70 % |  |
| **Follow-up after diagnosis** | No | Yes |  |
| **Diagnosis expense** | RMB 150 | RMB 0 |  |
| **Which option would you most prefer?**  **(tick one box)** |  |  |  |

**Question 4**

When you have the same disease and the same physical condition, there are three options to choose Doctor A or Doctor B and neither. Please choose the one you are most satisfied with from the following three options?

|  | **Doctor A** | **Doctor B** | **None** |
| --- | --- | --- | --- |
| **Diagnosis method** | Line up for face-to-face visits in outpatient clinics | AI rapid diagnosis |  |
| **Outpatient waiting time** | 20 min | 40 min |  |
| **Diagnosis time** | 30 min | 0 min |  |
| **Accuracy** | 70 % | 80 % |  |
| **Follow-up after diagnosis** | No | Yes |  |
| **Diagnosis expense** | RMB 150 | RMB 50 |  |
| **Which option would you most prefer?**  **(tick one box)** |  |  |  |

**Question 5**

When you have the same disease and the same physical condition, there are three options to choose Doctor A or Doctor B and neither. Please choose the one you are most satisfied with from the following three options?

|  | **Doctor A** | **Doctor B** | **None** |
| --- | --- | --- | --- |
| **Diagnosis method** | Line up for face-to-face visits in outpatient clinics | AI rapid initial diagnosis + doctor's diagnosis |  |
| **Outpatient waiting time** | 20 min | 60 min |  |
| **Diagnosis time** | 30 min | 15 min |  |
| **Accuracy** | 100 % | 80 % |  |
| **Follow-up after diagnosis** | Yes | No |  |
| **Diagnosis expense** | RMB 250 | RMB 0 |  |
| **Which option would you most prefer?**  **(tick one box)** |  |  |  |

**Question 6**

When you have the same disease and the same physical condition, there are three options to choose Doctor A or Doctor B and neither. Please choose the one you are most satisfied with from the following three options?

|  | **Doctor A** | **Doctor B** | **None** |
| --- | --- | --- | --- |
| **Diagnosis method** | AI rapid initial diagnosis + doctor's diagnosis | AI rapid initial diagnosis + doctor's diagnosis |  |
| **Outpatient waiting time** | 0 min | 40 min |  |
| **Diagnosis time** | 0 min | 15 min |  |
| **Accuracy** | 80 % | 60 % |  |
| **Follow-up after diagnosis** | No | No |  |
| **Diagnosis expense** | RMB 0 | RMB 50 |  |
| **Which option would you most prefer?**  **(tick one box)** |  |  |  |

**Question 7**

When you have the same disease and the same physical condition, there are three options to choose Doctor A or Doctor B and neither. Please choose the one you are most satisfied with from the following three options?

|  | **Doctor A** | **Doctor B** | **None** |
| --- | --- | --- | --- |
| **Diagnosis method** | AI rapid diagnosis | AI rapid initial diagnosis + doctor's diagnosis |  |
| **Outpatient waiting time** | 60 min | 80 min |  |
| **Diagnosis time** | 0 min | 15 min |  |
| **Accuracy** | 100 % | 90 % |  |
| **Follow-up after diagnosis** | No | No |  |
| **Diagnosis expense** | RMB 200 | RMB 100 |  |
| **Which option would you most prefer?**  **(tick one box)** |  |  |  |
