# Supplementary material for: Preferences for Artificial Intelligence Clinicians Before and During the COVID-19 Pandemic: Discrete Choice Experiment and Propensity Score Matching Study
Source: J Med Internet Res. 2021 Mar 2;23(3):e26997. doi: 10.2196/26997 (PMC7927951; doi:10.2196/26997)
Supplement: Multimedia Appendix 3 [file jmir_v23i3e26997_app3.docx]

**S2 Appendix PSM**

Propensity score matching (PSM) is a regression method which collect and set the respondents with similar basic situation for the treatment group and the control group, and prevalently applied in the study of impact factors and casual effects, such as a medical treatment, a policy-decision, or a case study. The implementation steps of PSM can be concluded as 5 steps: 1) propensity score estimation; 2) choose matching algorithm; 3) check overlap/common support; 4) matching quality/effect estimation; 5) sensitivity analysis.

- **Mathematical model**

The mathematical theory of PSM is primarily based on the Roy-Robin model. The most fundamental causal problem that has been hypothesized that the evaluation model needs to solve is whether the drug has an onset of patients’ condition, and whether the action of the drug caused the patient to get better or the patient has recovered by the self-healing ability or other reasons. In order to infer whether the treatment has affected the result, the most ideal way is to treat the patients or not, and then observe whether the patients’ condition has been improved. Thus, the patients’ own factors which lead to the conditional improvement can be excluded. The formula can be interpreted as:

$\tau_{i}=Y_{i}\left( 1 \right)-Y_{i}(0)$ (1)

Where the *Y* represents the potential outcome, $Y_{i}\left( 1 \right)$ represents the outcome of receiving the treatment, $Y_{i}(0)$ represents the outcome of refusing the treatment, and $\tau_{i}$ represents the individual treatment effect. Because of the counterfactual outcome and the potential small-size sample size, the mean of the sample then should be shed a light on. One of the average individual treatment effect is called average treatment effect on the treated and represented as $\tau_{ATT}$, and the formula of the average treatment effect on the treated can be defined as:

$\tau_{ATT}=E\left( \tau| D=1 \right)=E\left[ Y\left( 1 \right) | D=1 \right]-E\left[ Y\left( 0 \right) | D=1 \right]$ (2)

Where $E\left[ Y\left( 1 \right) | D=1 \right]$ is not used here since the bias can arise due to the outcome might also been affected by the factors that decide the treatment. Thus, we hypothesize that the difference arises between treatment group and control group only resulted from the treatment, then, the formula would be:

$E\left[ Y\left( 1 \right) | D=1 \right]-E\left[ Y\left( 0 \right) | D=0 \right]=\tau_{ATT}+E\left[ Y\left( 0 \right) | D=1 \right]-E[Y(0)|D=0]$ (3)

Where the self-selection bias has been defined as $E\left[ Y\left( 1 \right) | D=1 \right]-E\left[ Y\left( 0 \right) | D=0 \right]-\tau_{ATT.}$ And if the outcome is not correlated with the treatment, then the parameter can be identified here.

**Reference**

1. Caliendo M, Kopeinig S. Some practical guidance for the implementation of propensity score matching. Journal of Economic Surveys. 2008;22(1):31-72
