# Supplementary material for: Preferences for Artificial Intelligence Clinicians Before and During the COVID-19 Pandemic: Discrete Choice Experiment and Propensity Score Matching Study
Source: J Med Internet Res. 2021 Mar 2;23(3):e26997. doi: 10.2196/26997 (PMC7927951; doi:10.2196/26997)
Supplement: Multimedia Appendix 4 [file jmir_v23i3e26997_app4.docx]

### S3 Appendix RUM

- **Theoretical model**

The basic theoretical model for analyzing the data collected from DCE is the random utility model (RUM). This framework assumes that the respondent n is will prefer doctor a to doctor b only if:

$U_{na}>U_{nb} \forall a\neq b$ (1)

Moreover, the RUM defines that the total utility U can be divided as two parts. The first part is fixed utility or in another word, deterministic utility, which consist of the observable elements. The second part is the random utility, which includes the unobservable elements and potential errors, the formula of the utility of the individual n can be interpreted as:

$U_{n}=V_{n}+\varepsilon_{n}=\alpha_{1}+\beta_{1}x_{1n}+\beta_{2}x_{2n}+\ldots+\beta_{m}x_{mni}+\varepsilon_{n}$ (2)

Where *x* represents the observable elements and $\beta$ represents the strength of the patients’ preference for one thing. However, this kind of strength is not always directly observable.

- **Logit model basic principle**

When the strength of people’s preference is not evidently observed and quantified, the framework of probability is then needed to measure that how likely a respondent will choose something. In this case, the respondent will choose doctor a over doctor b can be defined as:

$P_{na}=\Pr\left[ U_{na}>U_{nb} \right] \forall a\neq b$ (3)

Then we put the formula of RUM into the probability framework, it will turn into

$P_{n}=Pr[\varepsilon_{na}-\varepsilon_{nb}>V_{nb}-V_{na}]$ (4)

With the logit model, the probability that a respondent chooses the object a will be interpreted as:

$P_{i}=\frac{exp(V_{i})}{\sum_{j=1}^{n}exp(V_{j})}$ (5)

**Reference**

1. WWH, USAID; CapacityPlus; THE WORLD BANK. How to Conduct a Discrete Choice Experiment for Health Workforce Recruitment and Retention in Remote and Rural Areas. In: A User Guide with Case Studies. World Health Organization, 20 Avenue Appia, 1211 Geneva 27, Switzerland: WHO Press; 2012.Available from: [https://www.who.int/hrh/resources/DCE_UserGuide_WEB.pdf?ua=1.](https://www.who.int/hrh/resources/DCE_UserGuide_WEB.pdf?ua=1)
