# Supplementary material for: Preferences for Artificial Intelligence Clinicians Before and During the COVID-19 Pandemic: Discrete Choice Experiment and Propensity Score Matching Study
Source: J Med Internet Res. 2021 Mar 2;23(3):e26997. doi: 10.2196/26997 (PMC7927951; doi:10.2196/26997)
Supplement: Multimedia Appendix 5 [file jmir_v23i3e26997_app5.docx]

**Table S1.** Results of the latent class model for respondents in the 2017 group.

| Attributes and levels | | Class 1 (n=228) | | | Class 2 (n=223) | | | Class 3 (n=77) | | |
| --- | --- | --- | --- | --- | --- | --- | --- | --- | --- | --- |
|  | | Coefficient | *P* value | Odds ratio (95% CI) | Coefficient | *P* value | Odds ratio (95% CI) | Coefficient | *P* value | Odds ratio (95% CI) |
|  | |  |  |  |  |  |  |  |  |  |
| **Diagnosis method** | | | | | | | | | | |
|  | Clinician | −0.355 | <.001 | Reference | 0.007 | .92 | Reference | 0.390 | <.001 | Reference |
|  | Artificial intelligence and clinician | 0.553 | <.001 | 2.479 (2.240-2.743) | 0.193 | .01 | 1.204 (1.039-1.394) | 0.155 | .17 | 0.791 (0.636-0.984) |
|  | Artificial intelligence | −0.199 | <.001 | 1.169 (1.060-1.289) | −0.200 | .008 | 0.813 (0.702-0.942) | −0.546 | <.001 | 0.392 (0.308-0.500) |
| **Outpatient waiting time (minutes)** | | | | | | | | | | |
|  | 0 | 0.302 | <.001 | Reference | 0.365 | .001 | Reference | 0.564 | <.001 | Reference |
|  | 20 | −0.090 | .25 | 0.676 (0.580-0.787) | 0.664 | <.001 | 1.349 (1.065-1.708) | 0.349 | .03 | 0.806 (0.594-1.095) |
|  | 40 | −0.124 | .11 | 0.653 (0.561-0.761) | 0.174 | .14 | 0.826 (0.657-1.039) | 0.135 | .41 | 0.651 (0.472-0.898) |
|  | 60 | 0.094 | .23 | 0.812 (0.697-0.946) | −0.393 | <.001 | 0.469 (0.374-0.587) | −0.648 | <.001 | 0.298 (0.207-0.429) |
|  | 80 | −0.181 | .02 | 0.617 (0.530-0.718) | −0.809 | <.001 | 0.309 (0.246-0.389) | −0.400 | .02 | 0.381 (0.271-0.536) |
| **Diagnosis time (minutes)** | | | | | | | | | | |
|  | 0 | 0.018 | .72 | Reference | 0.253 | <.001 | Reference | −0.113 | .33 | Reference |
|  | 15 | 0.004 | .94 | 0.986 (0.893-1.088) | −0.203 | .007 | 0.633 (0.547-0.733) | −0.217 | .07 | 0.902 (0.717-1.135) |
|  | 30 | −0.021 | .67 | 0.961 (0.871-1.061) | −0.050 | .501 | 0.738 (0.639-0.854) | 0.330 | .004 | 1.558 (1.252-1.938) |
| **Diagnosis accuracy (% accuracy)** | | | | | | | | | | |
|  | 60 | −0.173 | .03 | Reference | −2.754 | <.001 | Reference | −0.673 | <.001 | Reference |
|  | 70 | −0.207 | .009 | 0.967 (0.829-1.128) | -0.790 | <.001 | 7.129 (5.742-8.851) | −0.448 | .02 | 1.252 (0.877-1.787) |
|  | 80 | 0.252 | .001 | 1.530 (1.315-1.781) | −0.059 | .57 | 14.804 (12.082-18.140) | 0.027 | .87 | 2.014 (1.472-2.757) |
|  | 90 | −0.004 | .96 | 1.184 (1.015-1.380) | 1.195 | <.001 | 51.923 (41.340-65.216) | 0.178 | .28 | 2.341 (1.705-3.214) |
|  | 100 | 0.132 | .09 | 1.357 (1.164-1.581) | 2.408 | <.001 | 174.651 (129.781-235.034) | 0.916 | <.001 | 4.899 (3.631-6.611) |
| **Follow-up after diagnosis** | | | | | | | | | | |
|  | Yes | 0.183 | <.001 | Reference | 0.402 | <.001 | Reference | 0.236 | .002 | Reference |
|  | No | −0.183 | <.001 | 0.694 (0.653-0.737) | −0.402 | <.001 | 0.447 (0.406-0.493) | −0.236 | .002 | 0.623 (0.538-0.722) |
| **Diagnosis expenses (¥**^a^**)** | | | | | | | | | | |
|  | 0 | 0.201 | .03 | Reference | 1.114 | <.001 | Reference | 0.675 | <.001 | Reference |
|  | 50 | 0.108 | .22 | 0.912 (0.766-1.084) | 0.853 | <.001 | 0.770 (0.592-1.002) | 0.333 | .07 | 0.770 (0.592-1.002) |
|  | 100 | 0.017 | .85 | 0.832 (0.699-0.989) | 0.031 | 0.80 | 0.339 (0.266-0.432) | 0.045 | .81 | 0.339 (0.266-0.432) |
|  | 150 | 0.098 | .27 | 0.902 (0.759-1.073) | −0.219 | 0.08 | 0.264 (0.206-0.337) | 0.027 | .88 | 0.264 (0.206-0.337) |
|  | 200 | −0.124 | 0.17 | 0.723 (0.606-0.861) | −0.939 | <.001 | 0.128 (0.096-0.172) | −0.318 | .11 | 0.128 (0.096-0.172) |
|  | 250 | −0.299 | <.001 | 0.607 (0.509-0.723) | −0.840 | <.001 | 0.142 (0.109-0.185) | −0.762 | <.001 | 0.142 (0.109-0.185) |

^a^A currency exchange rate of ¥1=US $0.16 is applicable.

**Table S2.** Results of the latent class model for respondents in the 2020 group.

| Attributes and levels | | Class 1 (n=237) | | | Class 2 (n=254) | | | Class 3 (n=37) | | |
| --- | --- | --- | --- | --- | --- | --- | --- | --- | --- | --- |
|  | | Coefficient | *P* value | Odds ratio (95% CI) | Coefficient | *P* value | Odds ratio (95% CI) | Coefficient | *P* value | Odds ratio (95% CI) |
|  | |  |  |  |  |  |  |  |  |  |
| **Diagnosis method** | | | | | | | | | | |
|  | Clinician | 0.090 | .18 | Reference | −0.159 | <.001 | Reference | −0.220 | .34 | Reference |
|  | Artificial intelligence and clinician | 0.217 | .001 | 1.135 (0.997-1.293) | 0.538 | <.001 | 2.009 (1.826-2.211) | 0.075 | .73 | 1.343 (0.877-2.059) |
|  | Artificial intelligence | −0.307 | <.001 | 0.672 (0.587-0.769) | −0.379 | <.001 | 0.803 (0.731-0.883) | 0.145 | .52 | 1.442 (0.926-2.244) |
| **Outpatient waiting time (minutes)** | | | | | | | | | | |
|  | 0 | 0.609 | <.001 | Reference | −0.021 | .78 | Reference | −0.019 | .95 | Reference |
|  | 20 | 0.197 | .052 | 0.662 (0.543-0.807) | 0.377 | <.001 | 1.488 (1.287-1.721) | 0.207 | .504 | 1.254 (0.686-2.291) |
|  | 40 | −0.119 | .27 | 0.483 (0.391-0.596) | 0.024 | .75 | 1.046 (0.903-1.211) | 0.451 | .13 | 1.599 (0.901-2.838) |
|  | 60 | −0.176 | .09 | 0.456 (0.373-0.557) | −0.282 | <.001 | 0.770 (0.667-0.890) | −0.501 | .17 | 0.617 (0.308-1.238) |
|  | 80 | −0.512 | <.001 | 0.326 (0.268-0.397) | −0.098 | .18 | 0.925 (0.801-1.069) | −0.138 | .69 | 0.887 (0.454-1.735) |
| **Diagnosis time (minutes)** | | | | | | | | | | |
|  | 0 | −0.021 | .76 | Reference | −0.003 | .95 | Reference | 0.243 | .26 | Reference |
|  | 15 | −0.008 | .90 | 1.013 (0.889-1.154) | 0.006 | .91 | 1.009 (0.919-1.108) | −0.238 | .33 | 0.619 (0.386-0.992) |
|  | 30 | 0.029 | .66 | 1.051 (0.923-1.197) | −0.002 | .96 | 1.001 (0.911-1.099) | −0.005 | .98 | 0.781 (0.508-1.200) |
| **Diagnosis accuracy (% accuracy)** | | | | | | | | | | |
|  | 60 | −2.149 | <.001 | Reference | −0.272 | <.001 | Reference | −1.186 | .02 | Reference |
|  | 70 | −1.008 | <.001 | 3.130 (2.547-3.845) | −0.133 | .07 | 1.149 (0.996-1.326) | −0.917 | .047 | 1.308 (0.544-3.143) |
|  | 80 | −0.092 | .32 | 7.820 (6.522-9.376) | 0.060 | .41 | 1.394 (1.208-1.608) | 0.280 | .40 | 4.333 (2.285-8.215) |
|  | 90 | 1.027 | <.001 | 23.951 (19.729-29.078) | 0.119 | .11 | 1.478 (1.276-1.711) | 0.549 | .08 | 5.666 (3.117-10.301) |
|  | 100 | 2.223 | <.001 | 79.203 (61.423-102.129) | 0.226 | .002 | 1.645 (1.423-1.901) | 1.274 | <.001 | 11.709 (6.759-20.285) |
| **Follow-up after diagnosis** | | | | | | | | | | |
|  | Yes | 0.540 | <.001 | Reference | 0.066 | .03 | Reference | 0.260 | .11 | Reference |
|  | No | −0.540 | <.001 | 0.340 (0.310-0.373) | −0.066 | .03 | 0.876 (0.827-0.928) | −0.260 | .11 | 0.594 (0.436-0.810) |
| **Diagnosis expenses (¥**^a^**)** | | | | | | | | | | |
|  | 0 | 0.466 | <.001 | Reference | 0.425 | <.001 | Reference | 0.946 | .006 | Reference |
|  | 50 | 0.359 | .002 | 0.912 (0.766-1.084) | 0.136 | .10 | 0.749 (0.636-0.882) | 1.162 | <.001 | 1.241 (0.669-2.303) |
|  | 100 | 0.273 | .02 | 0.832 (0.699-0.989) | 0.213 | .012 | 0.809 (0.686-0.955) | −0.180 | .65 | 0.324 (0.152-0.694) |
|  | 150 | −0.010 | .93 | 0.902 (0.759-1.073) | −0.104 | .22 | 0.589 (0.500-0.694) | −0.154 | .68 | 0.333 (0.159-0.696) |
|  | 200 | −0.347 | .004 | 0.723 (0.606-0.861) | −0.145 | .08 | 0.565 (0.480-0.666) | −1.551 | .02 | 0.082 (0.025-0.272) |
|  | 250 | −0.740 | <.001 | 0.607 (0.509-0.723) | −0.524 | <.001 | 0.387 (0.327-0.458) | −0.222 | .59 | 0.311 (0.141-0.687) |

^a^A currency exchange rate of ¥1=US $0.16 is applicable.
